# A native phosphoglycolate salvage pathway of the synthetic autotrophic yeast *Komagataella phaffii*

**DOI:** 10.1093/femsml/uqad046

**Published:** 2023-12-11

**Authors:** Michael Baumschabl, Bernd M Mitic, Christina Troyer, Stephan Hann, Özge Ata, Diethard Mattanovich

**Affiliations:** Austrian Centre of Industrial Biotechnology, Vienna 1190, Austria; Department of Biotechnology, Institute of Microbiology and Microbial Biotechnology, University of Natural Resources and Life Sciences, Vienna 1190, Austria; Department of Biotechnology, Institute of Microbiology and Microbial Biotechnology, University of Natural Resources and Life Sciences, Vienna 1190, Austria; University of Natural Resources and Life Sciences, Vienna, Department of Chemistry, Institute of Analytical Chemistry, Vienna 1190, Austria; University of Natural Resources and Life Sciences, Vienna, Department of Chemistry, Institute of Analytical Chemistry, Vienna 1190, Austria; Austrian Centre of Industrial Biotechnology, Vienna 1190, Austria; University of Natural Resources and Life Sciences, Vienna, Department of Chemistry, Institute of Analytical Chemistry, Vienna 1190, Austria; Austrian Centre of Industrial Biotechnology, Vienna 1190, Austria; Department of Biotechnology, Institute of Microbiology and Microbial Biotechnology, University of Natural Resources and Life Sciences, Vienna 1190, Austria; Austrian Centre of Industrial Biotechnology, Vienna 1190, Austria; Department of Biotechnology, Institute of Microbiology and Microbial Biotechnology, University of Natural Resources and Life Sciences, Vienna 1190, Austria

**Keywords:** autotrophic, calvin cycle, CO2, capture, phosphoglycolate, synthetic, yeast

## Abstract

Synthetic autotrophs can serve as chassis strains for bioproduction from CO_2_ as a feedstock to take measures against the climate crisis. Integration of the Calvin–Benson–Bassham (CBB) cycle into the methylotrophic yeast *Komagataella phaffii* (*Pichia pastoris*) enabled it to use CO_2_ as the sole carbon source. The key enzyme in this cycle is ribulose-1,5-bisphosphate carboxylase/oxygenase (RuBisCO) catalyzing the carboxylation step. However, this enzyme is error prone to perform an oxygenation reaction leading to the production of toxic 2-phosphoglycolate. Native autotrophs have evolved different recycling pathways for 2-phosphoglycolate. However, for synthetic autotrophs, no information is available for the existence of such pathways. Deletion of *CYB2* in the autotrophic *K. phaffii* strain led to the accumulation of glycolate, an intermediate in phosphoglycolate salvage pathways, suggesting that such a pathway is enabled by native *K. phaffii* enzymes. ^13^C tracer analysis with labeled glycolate indicated that the yeast pathway recycling phosphoglycolate is similar to the plant salvage pathway. This orthogonal yeast pathway may serve as a sensor for RuBisCO oxygenation, and as an engineering target to boost autotrophic growth rates in *K. phaffii*.

## Teaser

A native phosphoglycolate salvage pathway was characterized in a synthetic autotrophic *K. phaffii* strain showing the robustness of the yeast's metabolism in responding to a synthetic pathway and its side reactions.

## Introduction

The Calvin–Benson–Bassham (CBB) cycle is responsible for the vast majority of carbon fixed on our planet. The key enzyme and rate limiting step of this cycle is ribulose-1,5-bisphosphate carboxylase/oxygenase (RuBisCO), which catalyzes the carbon dioxide fixation to ribulose-1,5-bisphosphate. With an average turn over number of around 3 s^−1^ RuBisCO is a very slow enzyme. Plants therefore produce huge amounts of this protein, with a RuBisCO content of around 30% to 50% of soluble protein (Feller et al. 2008). In addition, RuBisCO gets easily inhibited by a range of other sugar phosphates. These inhibitors can be released by the action of specific RuBisCO activases (Parry et al. 2008, Hauser et al. 2015).

Another aspect which reduces the efficiency of the RuBisCO protein is the fact that it also reacts with oxygen instead of CO_2_ which reduces the carbon fixation rate. The rate of this unfavorable oxygenation reaction varies between different types of RuBisCO. In general, the RuBisCO protein family can be subdivided into 4 types where type I and II are the predominant forms in organisms performing the CBB cycle. Type II proteins have often higher turnover numbers compared to type I proteins but lack their higher specificity to CO_2_ leading to an inverse correlation between the specificity and the turnover rate (Tcherkez et al. 2006).

**Figure 1.**
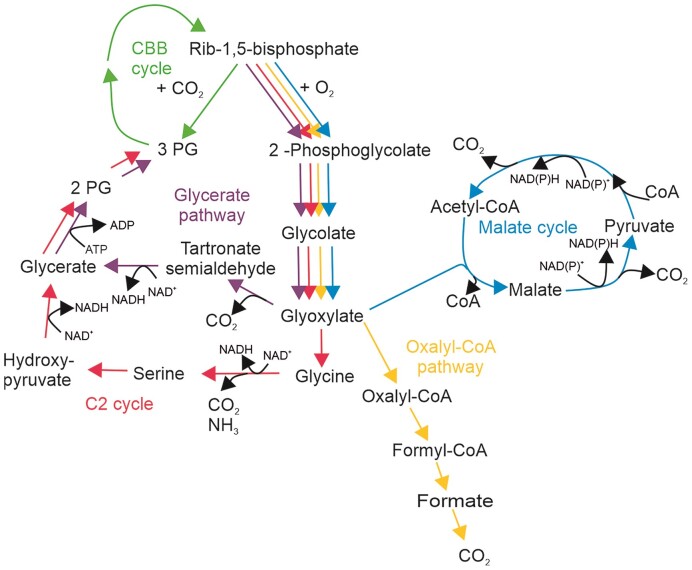
Overview of different phosphoglycolate salvage pathways: C2 cycle in red, glycerate pathway in purple, oxalyl CoA pathway in yellow and the malate cycle in blue. CBB: Calvin–Benson–Bassham cycle, 2-PG: 2-phosphoglycerate, 3-PG: 3-phosphoglycerate, Rib-1,5-bisphosphate: ribulose-1,5-bisphosphate, CoA: coenzyme A, PEP: phosphoenolpyruvate, OxAc: oxaloacetate.

Even though various organisms harboring the CBB cycle perform carbon concentrating mechanisms to improve their carbon capture efficiency (Keeley and Rundel 2003, Raven et al. 2008, Yeates et al. 2008, Cameron et al. 2013, Wang et al. 2015), the oxygenation reaction is always present to some degree. Oxygenation of ribulose 1,5-bisphosphate produces one molecule 3-phosphoglycerate (3-PG) and one molecule 2-phosphoglycolate. 2-phosphoglycolate is reported to be toxic in plants because it can inhibit at least two key enzymes of the central carbon metabolism, triose phosphate isomerase and phosphofructokinase (Hall et al. 1987, Flügel et al. 2017). Therefore, different types of organisms have evolved different pathways for recycling 2- phosphoglycolate (Fig. 1). In plants, most algae and cyanobacteria this is called the C2 cycle (Fig. 1, red line). It begins with the dephosphorylation of 2-phosphogycolate, followed by the oxidation into glyoxylate. Glyoxylate is then transaminated to glycine using either serine or glutamate as an amino group donor. Two molecules of glycine are then converted to serine in the mitochondria, releasing ammonia and carbon dioxide. The serine is then transported back to the peroxisomes, where it donates its amino group to another glyoxylate molecule. The deamination product, hydroxypyruvate, is then transported to the cytosol and reduced to glycerate. Finally, glycerate is phosphorylated to 3-phosphoglycerate, which allows it to re-enter the CBB cycle (Bauwe et al. 2010). The C2 cycle in plants consumes 3.5 ATP and 2 NADH equivalents and releases 0.5 molecules of CO_2_ per RuBisCO oxygenation (Walker et al. 2016). In cyanobacteria two different pathways responsible for the salvage of 2-phosphoglycolate have been identified besides the C2 cycle. In one of these pathways, the glycerate pathway, 2 molecules of glyoxylate are converted to tartronate semialdehyde, further reduced to glycerate and phosphorylated ending up in 3-PG avoiding the release of ammonia compared to the C2 cycle (Fig. 1, violet line). The other pathway catalyzes the complete oxidation of 2-phosphoglycolate to CO_2_ via oxalate and formate in combination with harvesting of energy in the form of NADH (Fig. 1, yellow line) (Eisenhut et al. 2006, 2008). In the chemolithotrophic bacterium *Cupriavidus necator* the glycerate pathway is the main contributor to the recycling of 2-phosphoglycolate but another pathway was identified, the malate cycle which fully oxidizes glycolate to CO_2_ (Fig. 1, blue line) (Claassens et al. 2020) while harvesting energy in the form of 2 molecules NADH per glycolate oxidation.

All organisms which naturally perform the CBB cycle perform at least one of the possible routes for recycling of 2-phosphoglycolate. Lately the CBB cycle was successfully integrated in the two model organisms *Escherichia coli* (Gleizer et al. 2019) and *Komagataella phaffii* (Gassler et al. 2020). In both approaches no specific pathway for the phosphoglycolate recycling was integrated. Cells were able to grow efficiently with CO_2_ as sole carbon source leading to the assumption that the strains came up with a phosphoglycolate salvage pathway based on native enzymes. Here we characterize this native phosphoglycolate salvage pathway in the synthetic autotrophic *K. phaffii* strain and show the robustness of yeast's metabolism in responding to a synthetic pathway and its side reactions.

## Results

### Are synthetic autotrophs sensitive to oxygen?

In our previous paper, we showed that a synthetic autotrophic *K. phaffii* strain was able to grow on CO_2_ as sole carbon source and methanol as energy source. This was achieved by blocking methanol assimilation via the xylulose-5-phosphate cycle as well as by the integration of six genes of the CBB cycle (Gassler et al. 2020). The key enzyme of the integrated CBB cycle is the RuBisCO enzyme which fixes the carbon dioxide to ribulose-1,5-bisphosphate, but also catalyzes the reaction with oxygen which reduces the efficiency of the CBB cycle for growth on CO_2_. Therefore, we wanted to evaluate the effect of the oxygen concentration on growth rate of this strain. Bioreactor cultivations with oxygen concentrations in the inlet gas between 2.5% and 20% were performed and growth and dissolved oxygen were monitored.

**Figure 2.**
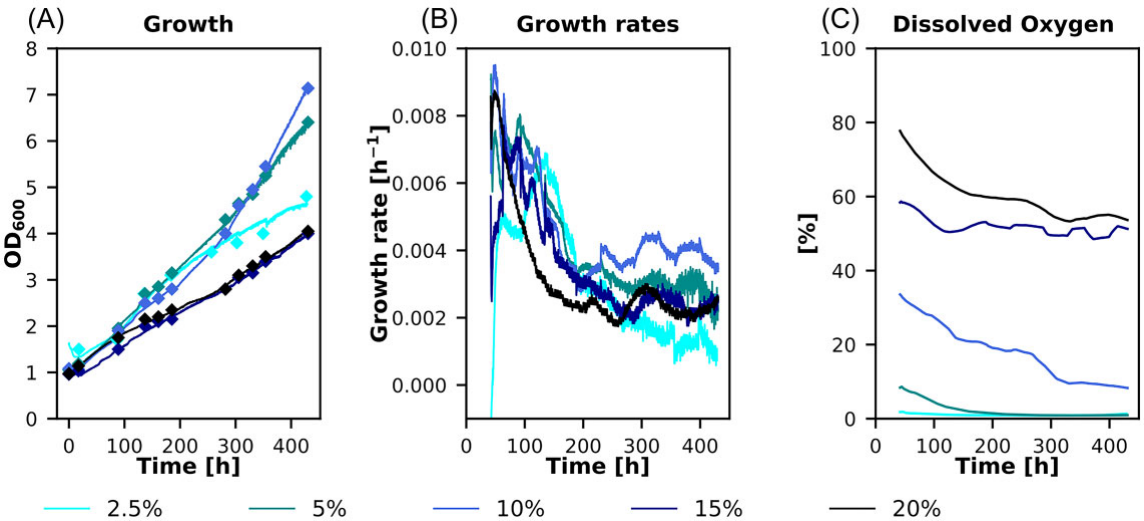
Bioreactor cultivations using different oxygen levels in the inlet air to test their influence on growth. (A) Diamonds: offline OD_600_ measurements and solid line online OD probe to monitor growth, (B) calculated growth rates and (C) dissolved oxygen concentrations of 5 different fermentations using oxygen concentrations in the inlet air from 2.5% to 20% v/v. Cultivations were performed at 30°C with a constant stirrer speed of 300 rpm and a CO_2_ concentration in the inlet air of 5%.

Different inlet oxygen concentrations resulted in a difference in the growth profile (Fig. 2A). The two lower oxygen concentrations of 5% and 10% resulted in faster growth compared to the higher oxygen levels in the inlet gas. The lowest oxygen concentration of 2.5% resulted in slightly lower growth compared to 5 and 10%. A similar picture was observed in an engineered version (Gassler et al. 2022) of this strain which enables it to reach faster autotrophic growth. Here the only difference in terms of oxygen sensitivity was a slightly increased oxygen demand of this strain (Supplementary Figure 1A), with an optimum oxygen concentration around 15%.

Many organisms performing the CBB cycle have come up with carbon concentrating mechanisms to locally increase CO_2_ concentration to reduce the rate of oxygenation. If these mechanisms are lacking in the synthetic autotrophic *K. phaffii*, how does it cope with the oxygenation reaction? While reducing the oxygen concentration improved the growth rate of the synthetic autotrophic yeast, it can also grow efficiently at ambient oxygen levels. This suggests that this synthetic autotroph is able to perform all reactions necessary to recycle 2-phosphoglycolate.

### A single gene deletion blocks phosphoglycolate salvage

Recently, we engineered this synthetic autotrophic *K. phaffii* for lactic acid production from CO_2_. To prevent the lactic acid consumption, we deleted *CYB2* encoding for an L-lactate cytochrome-c oxidoreductase which oxidizes lactate to pyruvate in the mitochondrial intermembrane space. Besides decreasing lactic acid reassimilation as intended, this knockout strain secreted glycolate into the medium under autotrophic conditions (Baumschabl et al. 2022). As shown in Fig. 1, glycolate is one the intermediate metabolites in the phosphoglycolate salvage pathways, therefore these results prompted us to hypothesize that there is a pathway responsible for the recycling of the formed phosphoglycolate where the oxidoreductase Cyb2 is involved.

**Figure 3.**
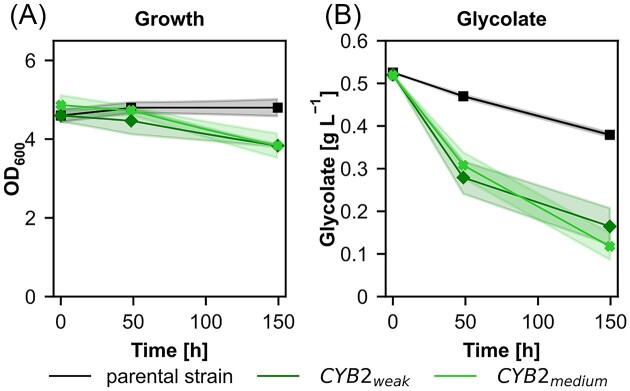
Cultivations using glycolate as sole carbon source to demonstrate that glycolate can be metabolized by *K. phaffii* and is suitable for further ^13^C tracer experiments (A) Growth and (B) glycolate concentrations in the supernatant of the synthetic autotrophic *K. phaffii* strain (parental strain) and *CYB2* overexpression strains using weak or medium strength promoters. Cultivations were performed at 30°C and ambient CO_2_ concentrations in the atmosphere. Solid lines indicate the mean and shades the standard deviation of three biological replicates.

To identify the possible route of 2-phosphoglycolate salvage formed by the oxygenation reaction of RuBisCO, we designed ^13^C tracer experiments with fully ^13^C labeled glycolate. Prior to the ^13^C tracer experiment, in order to make sure that glycolate can be metabolized by the cells and is suitable as a tracer metabolite, the synthetic autotrophic yeast strain and variants of this strain overexpressing *CYB*2 under the control of a medium or weak promoter were cultivated using glycolate as the only carbon source. None of the tested strains were able to grow on glycolate (Fig. 3A), but all strains could assimilate it. When *CYB2* was overexpressed, a higher assimilation rate was observed (Fig. 3B). These results confirmed that glycolate is assimilated in the metabolism and ^13^C from glycolate will therefore be incorporated demonstrating that glycolate can be used as a tracer metabolite for the ^13^C-labeling experiments.

### Identification of the native phosphoglycolate salvage pathway

**Figure 4.**
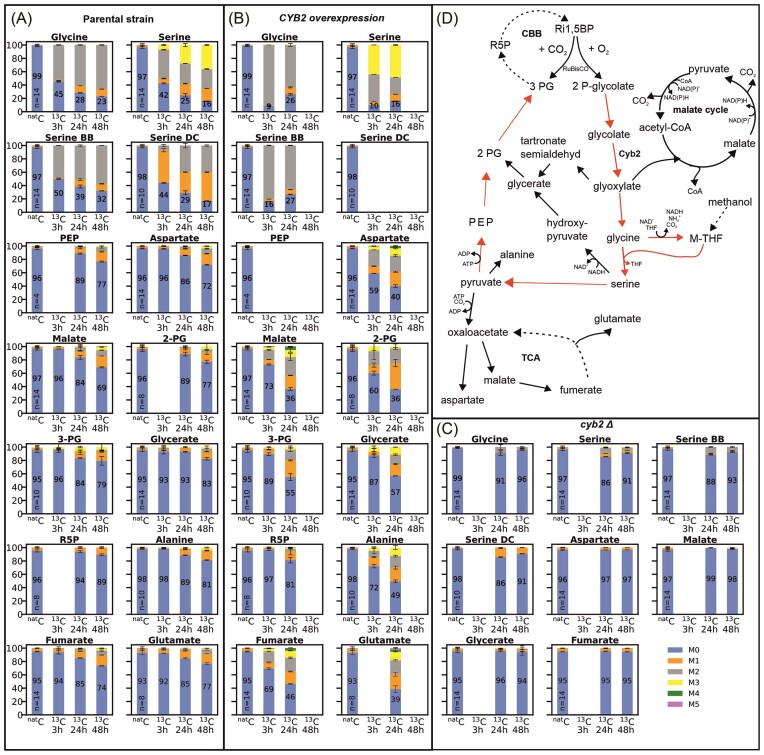
Carbon isotopologue distribution of intracellular metabolites resulting from tracer experiments using fully labeled ^13^C glycolate as carbon source to reveal the phosphoglycolate salvage pathway in the synthetic autotroph *K. phaffii* strain. Results of (A) parental strain, (B) *CYB2*_medium_ overexpression strain and (C) *CYB2* deletion strain. M0 to M5 corresponds to the number of ^13^C atoms present in the metabolite. The numbers in the blue bars indicate the isotopologue fraction of the M0 isotopologue of the respective sample. The number of biological replicates of the ^nat^C samples is indicated by ‘n=’ in the bars; 2 biological replicates were performed for all ^13^C labeled samples. Error bars represent the standard deviation. Serine BB denotes the amino acids backbone fragment including only the C1 and C2 carbon atom of serine. Serine DC denotes the decarboxylated molecule with the C1 carbon atom being cleaved off. For the parental strain all three, for the overexpression strain the first two and for the deletion strain the last two time points were measured. Data of the missing bars could not be evaluated as they did not match the quality criteria. (D) Phosphoglycolate salvage pathway in the synthetic autotrophic *K. phaffii* strain. Red arrows indicate the proposed pathway based on the measured metabolites’ carbon isotopologue distribution. Abbreviations: PEP: phosphoenolpyruvate, 2-PG: 2-phosphoglycerate, 3-PG: 3-phosphoglycerate, R5P: ribose 5-phosphate, Ru1,5BP: ribulose 1,5-bisphosphate, CBB: Calvin–Benson–Bassham cycle, M-THF: methylene tetrahydrofolate, TCA: tricarboxylic acid cycle, CoA: coenzyme A.

Glycolate can be efficiently assimilated by the autotrophic *K. phaffii* strain which is a prerequisite for its use as a tracer metabolite to further investigate the route of phosphoglycolate salvage. Therefore, three different strains, the parental autotrophic strain, a *CYB2* knockout strain and the *CYB2* overexpression strain using the medium strength promoter, were cultivated on fully labeled ^13^C glycolate for 48 hours. Samples were taken after 3, 24, and 48 hours for the determination of the isotopologue distribution of various intracellular metabolites using GC-TOFMS. If a metabolite was involved in the phosphoglycolate salvage pathway, it would show a decrease in the “M0” isotopologue (^12^C only) (blue bars in Fig. 4) and an increase in the higher mass isotopologues, showing that ^13^C from labeled glycolate is incorporated into the respective metabolite. Metabolites with the highest ^13^C content are supposed to be located at the start of a pathway. When interpreting the labeling data, it had to be considered that all strains produce ^12^C glycolate via oxygenation of ribulose 1,5-bisphosphate catalyzed by the expressed RuBisCO, which can reduce the incorporation of ^13^C atoms.

In the parental strain pronounced incorporation of ^13^C atoms into serine and glycine was observed already after 3 hours (Fig. 4A), indicating that most of the glycolate is recycled via glycine and not oxidized in the malate cycle. The ^13^C content of both metabolites increased until the end of cultivation. Neither pyruvate nor hydroxypyruvate could be analyzed with the applied GC-TOFMS method, hence the metabolic step following serine had to be deduced from labeling patterns of adjacent metabolites. Significant incorporation of ^13^C into PEP, 2-PG, 3-PG and glycerate was observed but to a lower extent compared to serine and glycine. Ribose 5-phosphate showed only a small fraction of ^13^C labeled isotopologues. Metabolites related to the TCA cycle such as malate and fumarate showed a similar labeling degree as the phosphorylated hydroxycarboxylic acids. Since malate and aspartate showed similar labeling patterns, the labeling pattern of both metabolites probably originates from oxaloacetate and not from the malate cycle. Two additional fragments of serine were evaluated: the backbone fragment (BB) corresponding to the amino acid backbone of serine containing the C1 and C2 carbon atom only and the decarboxylated fragment (DC) which corresponds to the decarboxylated serine molecule containing the C2 and C3 carbon atom. The two fragments showed significantly different labeling patterns. BB had a much higher fraction of the fully labeled isotopologue M2 compared to DC which showed a high fraction of M1, i.e. one labeled carbon atom. This finding indicates that especially at the beginning of the cultivation the methylene group of M-THF, which is used for the synthesis of serine from glycine, had a high ^12^C content. At later stages of the cultivation, the fraction of the M2 isotopologue of the DC fragment increased indicating that labeled glycine is used for M-THF synthesis.

In the strain overexpressing *CYB2* the labeling pattern of the metabolites was similar but in general a higher incorporation of ^13^C into the metabolites was observed (Fig. 4B). Already after 3 hours of cultivation on ^13^C glycolate, glycine and serine were nearly fully labeled. The ^13^C content was reduced at the next sampling time point (24 h), because nearly all ^13^C glycolate was consumed and more ^12^C glycolate produced from RuBisCO was present (Fig. S2). In 2-PG a higher incorporation of ^13^C was observed compared to 3-PG as well as glycerate, which showed similar labeling patterns. Again ribose 5-phosphate, the CBB intermediate evaluated here, showed only a small fraction of ^13^C labeled isotopologues. In addition, high ^13^C contents can be found in the TCA metabolites malate and fumarate as well as in aspartate and glutamate, deriving from oxaloacetate.

In contrast to the other two strains, the strain harboring the deletion of *CYB2* resulted in only minor incorporation of ^13^C into glycine and serine (Fig. 4C). All other analyzed metabolites did not show any significant incorporation of ^13^C-labeled carbon. As glycine and serine are weakly labeled in this strain, we hypothesized an alternative gene to *CYB2* catalyzing the same reaction to be present. One candidate gene was *DLD1* which shows similarities to *C. necator* glycerate dehydrogenase being responsible for the oxidation of glycolate to glyoxylate. However, an overexpression of this gene did not result in any significant glycolate consumption (Fig. S3).

**Figure 5.**
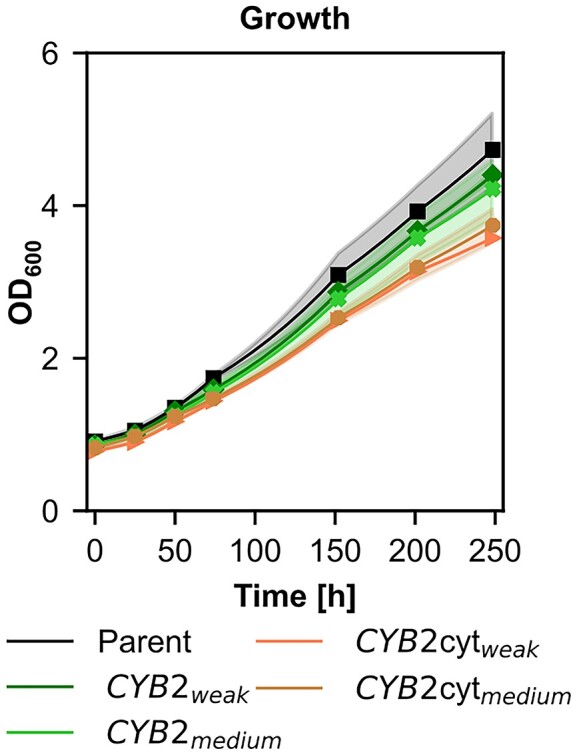
Cultivation of the *CYB2* overexpression strains compared to their parental strain to test whether the natural expression level of *CYB2* is a bottleneck for autotrophic growth. Cultivations were performed at 30°C and 5% CO_2_ concentrations in the atmosphere. Solid lines indicate the means, shades the standard deviation of the biological replicates.

The tracer experiments confirmed that *CYB2* plays a major role in the recycling of 2-phosphoglycolate produced by the oxygenation reaction of RuBisCO. Cyb2 is annotated as an L-(+)-lactate-cytochrome c oxidoreductase. Given the structural similarity of L-lactate and glycolate, the results can be interpreted such that Cyb2 also oxidizes glycolate to glyoxylate, taking over the role of glycolate oxidase and transferring the electrons to cytochrome *c* (Cunane et al. 2002). To evaluate if the natural activity of Cyb2 could lead to a bottleneck in phosphoglycolate recycling and therefore may limit the autotrophic growth rates, we cultivated *CYB2* overexpression strains using a medium and weak promoter under autotrophic conditions. We included also a cytosolic version of this protein to test if cytosolic location helps to boost the growth rate. None of the tested constructs could increase growth under autotrophic conditions showing the native expression levels of *CYB2* is sufficient for the cells to recycle the produced glycolate (Fig. 5). Deletion of the mitochondrial signal induced more stress and reduced growth rate even more in the cytosolic version of *CYB2*.

### The *CYB2* deletion strain can serve as RuBisCO oxygenation test platform

**Figure 6.**
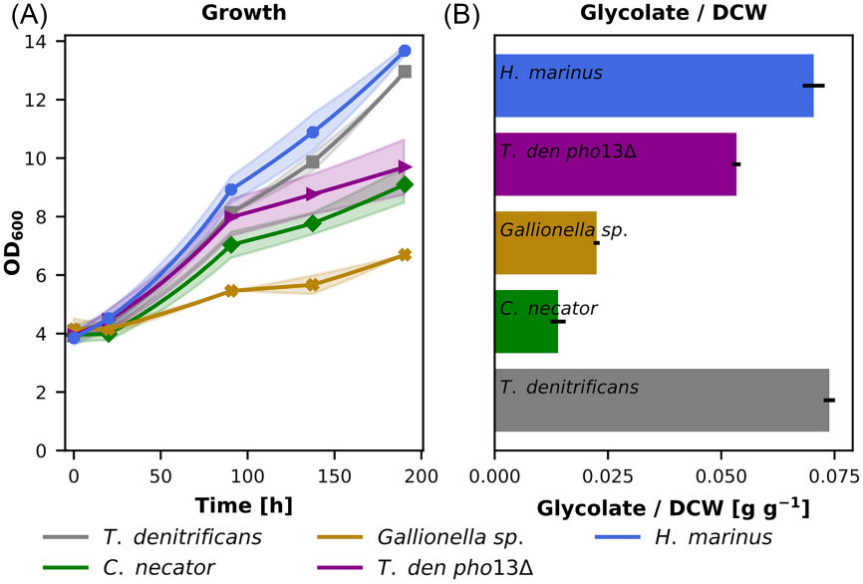
Testing the capabilities of the deletion of *CYB2* as oxygenation biosensor. Autotrophic *K. phaffii* strains using 4 different RuBisCO proteins, and a *pho13*Δ variant strain of the *T. denitrificans* RuBisCO strain were tested for (A) growth and (B) the ratio of glycolate to dry cell weight at the last time point. Cultivations were performed at 30°C and 5% CO_2_ in the atmosphere. Solid lines indicate the means, and shades (for A) and error bars (for B) the standard deviation of 3 biological replicates. The gycolate/biomass yield of *Gallionella sp*. RuBisCO might be interpreted carefully as the respective *K. phaffii* strain has a severe growth impairment on CO_2_.

Deletion of *CYB2* abolished the conversion of glycolate into glycine and serine and instead led to secretion of glycolate. We hypothesized that *cyb2Δ K. phaffii* strains with an intact CBB cycle could be used as RuBisCO oxygenation biosensor strains where the levels of glycolate secretion per biomass (glycolate/DCW) would correlate with the levels of oxygenation reaction of the chosen RuBisCO enzymes. Four different RuBisCO proteins were tested in the autotrophic *CYB2* deletion strain and growth as well as glycolate production were monitored (Fig. 6). The type II RuBisCO proteins of *T. denitrificans* and *Hydrogenovibrio marinus* resulted in fastest growth and similar glycolate production. The RuBisCO protein of *Gallionella sp*. could only barely facilitate growth on CO_2_ in our synthetic autotrophic strain. In addition, only small amounts of glycolate were produced. Switching to the type I RuBisCO of *C. necator*, which was tested together with the co-expression of its corresponding RuBisCO activase *CBBX* resulted in the lowest glycolate production per biomass (Fig. 6B). *C. necator* RuBisCO led, however, to a lower growth rate than the best performing RuBisCO variants. The lower specific glycolate production supports the concept that the *cyb2Δ* strain can serve as an indicator for the specificity of each RuBisCO (Table 1). Here, it should be noted that a higher specificity does not necessarily reflect a more efficient growth on CO_2_, but rather a lower sensitivity of the CBB cycle to oxygen.

**Table 1.** Overview of kinetic parameters of the RuBisCO proteins used in this study (measured using in vitro assays). K_M_: Michaelis Menten constant for CO_2_. k_cat_: turnover number for CO_2_. S_c/o_: CO_2_: O_2_ specificity factor.

Species	Oligomer	K_M_ [µM]	k_cat_ [s^−1^]	S_C/O_	Ref
*Thiobacillus. denitrificans cbbM*	L_2_	256	3.5	14	(Hernandez et al. 1996)
*Gallionella sp*.	L_2_	276	22.2	10	(Davidi et al. 2020)
*Hydrogenovibrio marinus*	L_2_	162	15.6	20	(Davidi et al. 2020)
*Cupriavidus necator cbbL/S*	L_8_S_8_	34	2.5	85	(Satagopan and Tabita 2016)

Also, a *PHO13* knockout strain was included in this series of experiments. This gene was the best BLAST hit of *K. phaffii* genes against *Arabidopsis thaliana PGLP1*, a 2-phosphoglycolate phosphatase. The deletion of this potential phosphoglycolate phosphatase resulted in a reduced growth rate compared to its parental strain. Glycolate production was also reduced, but both were not completely abolished, indicating that more than one phosphatase is acting on 2-phosphoglycolate in *K. phaffii*.

## Material and methods

### Strain generation

The host strain used in this study was the synthetic autotroph *K. phaffii* strain published by Gassler et al. (Gassler et al. 2020). The assembly of all DNA repair templates and over-expression cassettes were done using the Golden *Pi*CS cloning system (Prielhofer et al. 2017) and transformed using the CRISPR-Cas9 system (Gassler et al. 2019). Switching of the RuBisCO protein was done by deleting first the RuBisCO protein of the parental strain followed by the integration of the new RuBisCO protein. Successfully transformed clones were verified by colony PCR. All strains used in this work are given in Table 2.

**Table 2.** Overview of the strains used in this study.

Name	Genotype	Reference
Parental strain	CBS7435 *aox1Δ*::p*AOX1*_*TDH3* + p*FDH1*_*PRK* + p*ALD4*_*PGK1 das1*Δ::p*DAS1*_RuBisCO + p*PDC1*_*groEL* + p*RPP1B*_*groES das2*Δ::p*DAS2*_*TLK1* + p*RPS2*_*TPI1*	(Gassler et al. 2020)
Eng strain	Parent (PRK 5 C > G)	(Gassler et al. 2022)
*cyb2Δ*	Parent + *cyb2Δ*	This work
*CYB2_medium_*	Parent + p*MDH*3 *CYB2*	This work
*CYB2_weak_*	Parent + p*PDC1 CYB2*	This work
*T. den pho13Δ*	Parent + *cyb2Δ* + *pho13Δ*	This work
*Gallionella sp*.	Parent RuBisCO (*T denitrificans*):: RuBisCO (*Gallionella sp*.)	This work
	Parent RuBisCO (*T denitrificans*):: RuBisCO (*H. marinus*)	This work
	Parent RuBisCO*Δ* + p*DAS1 CbbL* + p*FDH1 CbbS* + p*ALD4 CbbX*	This work
*CYB2(cyt)_medium_*	Parent + p*MDH3 CYB2(del1_117)*	This work
*CYB2(cyt)_weak_*	Parent + p*PDC1 CYB2(del1_117)*	This work
*cyb2Δ DLD1_medium_*	*cyb2Δ +* p*MDH3 DLD1*	This work
*cyb2Δ DLD1_weak_*	*cyb2Δ +* p*LAT1 DLD1*	This work

### Shake flask cultivations

All strains were tested using shake flask cultures. As a first step a preculture on YPG (yeast extract 10 g L^−1^, soy peptone 20 g L^−1^, glycerol 20 g L^−1^) was performed overnight at 30°C and 180 rpm. Afterwards optical density was measured at 600 nm and the number of cells needed to inoculate the main culture (OD of 1, 4 or 20, respectively) was harvested, washed twice with water and resuspended in phosphate buffered Yeast Nitrogen Base Without Amino Acids (YNB) (3.4 g L^−1^, pH 6, 10 g L^−1^ (NH_4_)_2_SO_4_ as nitrogen source and 0.5% (vol/vol) methanol at the beginning as energy source). For the experiments using glycolate as carbon source additionally 0.5 g L^−1^ (for the pre-tests) or 1 g L^−1^ glycolic acid were added. All experiments using glycolate as carbon source were incubated at 30°C, 180 rpm and ambient CO_2_ concentrations. All experiments where cells were grown autotrophically were incubated at 30°C, 180 rpm, and 5% CO_2_ in the atmosphere. For all experiments, methanol concentrations were measured on the next day and adjusted to 1%. Afterwards samples were taken regularly and optical density, methanol and glycolic acid concentrations were measured. Water was added to correct the culture volume for evaporation.

### HPLC measurements

HPLC measurements to determine methanol and glycolic acid concentrations, were done according to an already published workflow (Baumschabl et al. 2022).

### Dry cell weight determination

Glass vials were incubated at 105°C for at least 24 hours, cooled down in the desiccator and weighed. Ten mL of culture were harvested by centrifugation, washed twice with water, transferred into the pre-weighed glass vials and incubated at 105°C until the cells were fully dried. After cooling down in the desiccator, the glass vials including the dried cells were weighed again and the dry cell weight was calculated.

### Labeling experiments

To determine the route of the phosphoglycolate salvage pathway, ^13^C labeling experiments were performed. The protocol was similar to the other shake flask cultivations using a starting OD of 20 but fully ^13^C labeled glycolic acid was used (Merck product no. 604011) for all ^13^C cultures and unlabeled glycolic acid with a natural isotopologe distribution for all ^nat^C cultures. Samples were taken at 2, 24, and 48 hours: 500 µL for the determination of optical density, the remaining glycolic acid as well as methanol concentration, and 3 mL for metabolic sampling & GC-TOFMS isotopologue analysis.

### Metabolic sampling & GC-TOFMS isotopologue analysis

The metabolomics workflow was performed as described by Mitic et al. (2023) based on the method of Mairinger et al. 2015.

In brief, the cell suspension was rapidly quenched in a 4-fold volume of 60% methanol, 125 mmol L^−1^ TRIS-HCl, 55 mmol L^−1^ NaCl at pH 8.2 and –27°C (Mattanovich et al. 2017) and filtered through a cellulose acetate filter after vortexing the mixture for 4 s. After washing the cells on the filter with 60% methanol, the filters were stored at –70°C. The consecutive boiling ethanol extraction was performed with 4 mL 75% ethanol at 85°C. After centrifugation the supernatants with the extracted metabolites were evaporated until complete dryness with a vacuum centrifuge before reconstitution in 1 mL H_2_O.

For the GC-TOFMS measurements, two methods were applied to cover all needed metabolites in the linear range of the instrument. Automated just-in-time derivatization prior to sample injection was employed to stabilize metabolites and reduce their boiling point. Ethoximation followed by trimethylsilylation was combined with splitless injection and chemical ionization for the analysis of the phosphorylated metabolites and sugar compounds as well as other intracellular metabolites of low abundance (Mairinger et al. 2015). *Tert*-butylsilylation was combined with 1:50 split injection and electron ionization for the analysis of organic acids and amino acids, a method which offers some positional information due to specific fragmentation patterns of the amino acids (Zamboni et al. 2009). The methods used for specific metabolites and samples are listed in Supplementary information Table S1.

For the evaluation of ^13^C incorporation into the metabolites isotopologue distribution analysis was conducted. The extracted ion chromatograms of all carbon isotopologues of a target analyte were integrated (evaluated mass/charge ratios listed in (Mitic et al. 2023)). The peak areas were corrected with the software ICT correction toolbox v.0.04 for the contribution of other heavy isotopes except ^13^C, as well as the contribution of the natural ^13^C abundance of the derivatization agent. The carbon isotopologue fractions were calculated as follows:


(1) \begin{eqnarray*}
{{Carbon\,\,isotopologue\,\,fraction}_i} = \frac{{{A_i}}}{{\sum\nolimits_{i = 0}^n {{A_i}} }}
\end{eqnarray*}

*n* = number of carbon atoms in the metabolite, *A_i_* = ICT corrected peak area of isotopologue i, i.e. an isotopologue containing i numbers of ^13^C atoms

For some metabolites such as the amino acids multiple fragments and/or adducts of both measurement methods display the carbon distribution. For data evaluation, the fragment or adduct was chosen based on the trueness of the ^nat^C control sample of the respective sequence (chosen method/fragment/adduct listed in Supplementary information Table S1). As the labeling experiments were conducted in biological duplicates, the displayed error bars stem from the standard deviation of duplicates multiplied with the correction factor 1.253314 to compensate for the small sample size (Roesslein et al. 2007). For the ^nat^C control samples average and standard deviation of all data are displayed.

### Bioreactor cultivations

Bioreactor cultivations were performed using 1.0 L DASGIP reactors (Eppendorf). Fermentations were performed using YNB with 10 g L^−1^ (NH_4_)_2_SO_4_ as the nitrogen source, buffered at pH 6 using 100 mmol L^−1^ phosphate buffer, 0.5% (vol/vol) methanol at the beginning as energy source, at 30°C and constant stirrer speed of 300 rpm. Different oxygen concentrations of 5, 10, 15 and 20%, respectively in the inlet gas flow were used to vary the available amount of oxygen, and CO_2_ concentrations were kept constant at 5%.

From an overnight YPG preculture the amount of cells to inoculate 500 mL of culture with an OD of 1 were harvested, washed twice with water and transferred into the bioreactors. The first sample was taken on the next day and methanol concentrations were adjusted to 1% (vol/vol). Afterwards samples were taken daily, and optical density and the methanol concentrations were measured. Every other day the methanol concentrations were adjusted to 1% (vol/vol).

## Discussion

Previously we were able to engineer the methylotrophic yeast *K. phaffii* to use CO_2_ as its sole carbon source by the integration of the CBB cycle. The carbon fixation step in this cycle is catalyzed by the RuBisCO enzyme. This enzyme also tends to perform the oxygenation reaction resulting in a loss of carbon fixation efficiency. In this study we characterized our synthetic autotrophic strains in terms of oxygen tolerance and tried to identify the pathway used for phosphoglycolate regeneration in this yeast strain.

Varying the oxygen concentration in the inlet air showed a significant influence on the growth rate of the autotrophic yeast strains, indicating an optimum dissolved oxygen concentration of around 15% (Fig. 2C and Supplementary Figure 1C). Reducing the oxygen levels helped the cells to grow faster, probably because of a reduction in the oxygenation reaction of RuBisCO. However, at least 5% oxygen in the inlet air are needed for efficient growth. Otherwise not enough oxygen is available for sufficient methanol oxidation providing energy for the cells, resulting in reduced growth rates as seen for the cultivations at 2.5% oxygen (Fig. 2). In the faster growing engineered version of this strain the optimal oxygen concentration is increased, probably due to the faster accumulation of biomass and therefore higher energy demand of the cells. A similar effect of a reduction of the oxygen concentration is also reported in microalgae (Kazbar et al. 2019), whereas in contrast most plants do not grow better under reduced oxygen conditions (Tisserat et al. 2002).

To get a deeper understanding of the pathways used to recycle the 2-phosphoglycolate produced via the oxygenation reaction of RuBisCO, a ^13^C tracer experiment using fully labeled glycolate was performed. The metabolites which showed early incorporation of ^13^C atoms were glycine and serine. This indicates that most of the phosphoglycolate is recycled and not fully oxidized to CO_2_. The further flux of the label is probably via deamination of serine to pyruvate, which is the branch point where the label enters the TCA cycle. Evaluation of the labeled metabolites led to the conclusion that glycolate is metabolized through pyruvate—PEP–2-PG and 3-PG, the latter reentering the CBB cycle (Fig. 4D). The *CYB2* overexpression strain showed a significantly higher incorporation of label into 2-PG compared to 3-PG and glycerate supporting the proposed route and dismissing the route present in plants via serine-hydroxypyruvate and glycerate. *CYB2* also serves as the major responsible gene for the oxidation of glycolate to glyoxylate, as the deletion of it blocked the phosphoglycolate recycling pathway almost completely and led to the secretion of glycolate, while in the native and overexpression strains no glycolate was secreted. These results indicate that native *CYB2* expression is already sufficient to enable a phosphoglycolate salvage pathway rate allowing efficient growth under autotrophic conditions.

As the deletion of *CYB2* leads to higher glycolate secretion, we tested whether a *cyb2Δ* strain can be used as a sensor strain for the oxygenation rate. We overexpressed different RuBisCO proteins and calculated the glycolate/biomass yield as an indicator for the specificity of RuBisCO for CO_2_ over oxygen. As expected, the choice of RuBisCO is a key factor for efficient autotrophic growth in the synthetic autotrophic *K. phaffii*. In general, the type II RuBisCO proteins led to faster growth compared to the type I protein tested. Only the type II protein from a *Gallionella sp.*, for which the highest turnover number so far was reported (Davidi et al. 2020), was not able to facilitate growth on CO_2_ in our synthetic autotrophic strain. Since the strain using this RuBisCO protein barely grows, the resulting glycolate production has to be taken with a grain of salt. Overall, the oxygenation test platform clearly revealed the difference of specificity between type I and II RuBisCO proteins. Both type II proteins enabling growth on CO_2_ resulted in an approximately 5 times higher glycolate production compared to the type I protein tested. This fits well to specificity data measured using *in vitro* assays (Table 1). Testing different RuBisCO proteins with varying oxygen concentrations might give more information about the robustness of the biosensor strain as well as the relationship between specificity and glycolate yield.

Similar to plants the recycling of 2-phosphoglycolate in the synthetic autotrophic *K. phaffii* releases 0.5 mol of previously captured carbon in the form of CO_2_ and additionally 0.5 mol ammonia per mol 2-phosphoglycolate. Both lead to a loss of invested energy. In addition, at least one molecule of ATP is needed during the recycling process in the step from pyruvate to PEP. However, the route via pyruvate and PEP is slightly more energy efficient compared to the hydroxypyruvate route since only one ATP contrary to one NADH has to be invested. In plants, the introduction of the glycerate pathway (Kebeish et al. 2007) or the malate cycle (Maier et al. 2012, South et al. 2019) into chloroplasts could improve the crop yields, making these pathways interesting engineering targets to improve the growth rate of the synthetic autotrophic *K. phaffii*.

With this work we could prove that balancing the available amount of oxygen can help to improve growth of the synthetic autotrophic *K. phaffii*. We were also able to solve the open question of how synthetic autotrophs can deal with the by-product of the oxygenation reaction and show how versatile a cell's metabolism can be in responding to newly formed substances and their toxic effects, such as 2-phosphoglycolate from the RuBisCO side reaction. As a next step, other more efficient glycolate salvage pathways like the glycerate pathway could be integrated into our strains to evaluate if they can further improve autotrophic growth rates.

## Supplementary Material

uqad046_Supplemental_File

## References

[bib1] Baumschabl M, Ata O, Mitic BM et al. Conversion of CO_2_ into organic acids by engineered autotrophic yeast. Proc Natl Acad Sci USA. 2022;119:e2211827119. 10.1073/pnas.2211827119/. 36383601 PMC9704707

[bib2] Bauwe H, Hagemann M, Fernie AR. Photorespiration: players, partners and origin. Trends Plant Sci. 2010;15:330–6. 10.1016/j.tplants.2010.03.006. 20403720

[bib3] Cameron JC, Wilson SC, Bernstein SL et al. XBiogenesis of a bacterial organelle: the carboxysome assembly pathway. Cell. 2013;155:1131. 10.1016/j.cell.2013.10.044. 24267892

[bib4] Claassens NJ, Scarinci G, Fischer A et al. Phosphoglycolate salvage in a chemolithoautotroph using the Calvin cycle. Proc Natl Acad Sci. 2020;117:22452–61. 10.1073/pnas.2012288117. 32820073 PMC7486775

[bib5] Cunane LM, Barton JD, Chen ZW et al. Crystallographic study of the recombinant flavin-binding domain of baker's yeast flavocytochrome b2: comparison with the intact wild-type enzyme. Biochemistry. 2002;41:4264–72. 10.1021/bi0119870. 11914072

[bib6] Davidi D, Shamshoum M, Guo Z et al. Highly active rubiscos discovered by systematic interrogation of natural sequence diversity. EMBO J. 2020;39:e104081. 10.15252/embj.2019104081. 32500941 PMC7507306

[bib7] Eisenhut M, Kahlon S, Hasse D et al. The plant-like C2 glycolate cycle and the bacterial-like glycerate pathway cooperate in phosphoglycolate metabolism in cyanobacteria. Plant Physiol. 2006;142:333–42. 10.1104/pp.106.082982. 16877700 PMC1557606

[bib8] Eisenhut M, Ruth W, Haimovich M et al. The photorespiratory glycolate metabolism is essential for cyanobacteria and might have been conveyed endosymbiontically to plants. Proc Natl Acad Sci. 2008;105:17199–204. 10.1073/pnas.0807043105. 18957552 PMC2579401

[bib9] Feller U, Anders I, Mae T. Rubiscolytics: fate of Rubisco after its enzymatic function in a cell is terminated. J Exp Bot. 2008;59:1615–24. 10.1093/jxb/erm242. 17975207

[bib10] Flügel F, Timm S, Arrivault S et al. The photorespiratory metabolite 2-phosphoglycolate regulates photosynthesis and starch accumulation in Arabidopsis. Plant Cell. 2017;29:2537–51. 10.1105/tpc.17.00256. 28947491 PMC5774572

[bib11] Gassler T, Baumschabl M, Sallaberger J et al. Adaptive laboratory evolution and reverse engineering enhances autotrophic growth in *Pichia pastoris*. Metab Eng. 2022;69:112–21. 10.1016/j.ymben.2021.11.007. 34800702

[bib12] Gassler T, Heistinger L, Mattanovich D et al. CRISPR/Cas9-mediated homology-directed genome editing in *Pichia pastoris*. Methods Mol Biol. 2019;1923:211–25. 10.1007/978-1-4939-9024-5_9. 30737742

[bib13] Gassler T, Sauer M, Gasser B et al. The industrial yeast *Pichia pastoris* is converted from a heterotroph into an autotroph capable of growth on CO_2_. Nat Biotechnol. 2020;38:210–6. 10.1038/s41587-019-0363-0. 31844294 PMC7008030

[bib14] Gleizer S, Ben-Nissan R, Bar-On YM et al. Conversion of *Escherichia coli* to Generate All Biomass Carbon from CO_2_. Cell. 2019;179:1255–63. e12. 10.1016/j.cell.2019.11.009. 31778652 PMC6904909

[bib15] Hall NP, Kendall AC, Lea PJ et al. Characteristics of a photorespiratory mutant of barley (*Hordeum vulgare* L.) deficient in phosphogly collate phosphatase. Photosynth Res. 1987;11:89–96. 10.1007/BF00117676. 24435465

[bib16] Hauser T, Popilka L, Hartl FU et al. Role of auxiliary proteins in Rubisco biogenesis and function. Nat Plants. 2015;1:15065. 10.1038/nplants.2015.65. 27250005

[bib17] Hernandez JM, Baker SH, Lorbach SC et al. Deduced amino acid sequence, functional expression, and unique enzymatic properties of the form I and form II ribulose bisphosphate carboxylase/oxygenase from the chemoautotrophic bacterium *Thiobacillus denitrificans*. J Bacteriol. 1996;178:347–56. 10.1128/jb.178.2.347-356.1996. 8550452 PMC177664

[bib18] Kazbar A, Cogne G, Urbain B et al. Effect of dissolved oxygen concentration on microalgal culture in photobioreactors. Algal Research. 2019;39:101432. 10.1016/j.algal.2019.101432.

[bib19] Kebeish R, Niessen M, Thiruveedhi K et al. Chloroplastic photorespiratory bypass increases photosynthesis and biomass production in *Arabidopsis thaliana*. Nat Biotechnol. 2007;25:593–9. 10.1038/nbt1299. 17435746

[bib20] Keeley JE, Rundel PW. Evolution of cam and C4 carbon-concentrating mechanisms. Int J Plant Sci. 2003;164:S55–77. 10.1086/374192.

[bib21] Maier A, Fahnenstich H, von Caemmerer S et al. Transgenic introduction of a glycolate oxidative cycle into *A. thaliana* chloroplasts leads to growth improvement. Front Plant Sci. 2012;3:38. 10.3389/fpls.2012.00038. 22639647 PMC3355595

[bib22] Mairinger T, Steiger M, Nocon J et al. Gas chromatography-quadrupole time-of-flight mass spectrometry-based determination of isotopologue and tandem mass isotopomer fractions of primary metabolites for ^13^C-metabolic flux analysis. Anal Chem. 2015;87:11792–802. 10.1021/acs.analchem.5b03173. 26513365

[bib23] Mattanovich M, Russmayer H, Scharl-Hirsch T et al. Metabolomics of *Pichia pastoris*: impact of buffering conditions on the kinetics and nature of metabolite loss during quenching. FEMS Yeast Res. 2017;17:fox016. 10.1093/femsyr/fox016. 28334329

[bib36_532_145323] Mitic BM, Troyer C, Lutz L et al. The oxygen-tolerant reductive glycine pathway assimilates methanol, formate and CO2 in the yeast Komagataella phaffii. Nat Commun. 2023; 14: 7754. 10.1038/s41467-023-43610-7. 38012236 PMC10682033

[bib24] Parry MAJ, Keys AJ, Madgwick PJ et al. Rubisco regulation: a role for inhibitors. J Exp Bot. 2008;59:1569–80. 10.1093/jxb/ern084. 18436543

[bib25] Prielhofer R, Barrero JJ, Steuer S et al. GoldenPiCS: a Golden Gate-derived modular cloning system for applied synthetic biology in the yeast *Pichia pastoris*. BMC Syst Biol. 2017;11:123. 10.1186/s12918-017-0492-3. 29221460 PMC5723102

[bib26] Raven JA, Cockell CS, De L et al. The evolution of inorganic carbon concentrating mechanisms in photosynthesis. Philosoph Transact Royal Soc B: Biolog Sci. 2008;363:2641–50. 10.1098/rstb.2008.0020. PMC2606764 18487130

[bib27] Roesslein M, Wolf M, Wampfler B et al. A forgotten fact about the standard deviation. Accredit Qual Assur. 2007;12:495–6. 10.1007/s00769-007-0285-2.

[bib28] Satagopan S, Tabita FR. RubisCO selection using the vigorously aerobic and metabolically versatile bacterium *Ralstonia eutropha*. FEBS J. 2016;283:2869–80. 10.1111/febs.13774. 27261087 PMC4975643

[bib29] South PF, Cavanagh AP, Liu HW et al. Synthetic glycolate metabolism pathways stimulate crop growth and productivity in the field. Science. 2019;363:eaat9077. 10.1126/science.aat9077. 30606819 PMC7745124

[bib30] Tcherkez GGB, Farquhar GD, Andrews TJ. Despite slow catalysis and confused substrate specificity, all ribulose bisphosphate carboxylases may be nearly perfectly optimized. Proc Natl Acad Sci. 2006;103:7246–51. 10.1073/pnas.0600605103. 16641091 PMC1464328

[bib31] Tisserat B, Vaughn S, Silman R. Influence of modified oxygen and carbon dioxide atmospheres on mint and thyme plant growth, morphogenesis and secondary metabolism in vitro. Plant Cell Rep. 2002;20:912–6. 10.1007/s00299-001-0428-6.

[bib32] Walker BJ, Vanloocke A, Bernacchi CJ et al. The costs of photorespiration to food production now and in the future. Annu Rev Plant Biol. 2016;67:107–29. 10.1146/annurev-arplant-043015-111709. 26865340

[bib33] Wang Y, Stessman DJ, Spalding MH. The CO_2_ concentrating mechanism and photosynthetic carbon assimilation in limiting CO_2_: how Chlamydomonas works against the gradient. Plant J. 2015;82:429–48. 10.1111/tpj.12829. 25765072

[bib34] Yeates TO, Kerfeld CA, Heinhorst S et al. Protein-based organelles in bacteria: carboxysomes and related microcompartments. Nat Rev Microbiol. 2008;6:681–91. 10.1038/nrmicro1913. 18679172

[bib35] Zamboni N, Fendt SM, Rühl M et al. ^13^C-based metabolic flux analysis. Nat Protoc. 2009;4:878–92. 10.1038/nprot.2009.58. 19478804

